# Supplementary material for: Quantitative three-dimensional imaging of chemical short-range order via machine learning enhanced atom probe tomography
Source: Nat Commun. 2023 Nov 16;14:7410. doi: 10.1038/s41467-023-43314-y (PMC10654683; doi:10.1038/s41467-023-43314-y)
Supplement: Supplementary file 1 — Supplementary Information [file 41467_2023_43314_MOESM1_ESM.pdf]

Supplementary information for

Quantitative three-dimensional imaging of chemical short-range order via machine learning enhanced atom probe tomography

Yue Li[1,], Ye Wei[1], Zhangwei Wang[2,*], Xiaochun Liu[3], Timoteo Colnaghi[4], Liuliu Han[1], Ziyuan Rao[1], Xuyang Zhou[1], Liam Huber[1], Raynol Dsouza[1], Yilun Gong[1], Jörg Neugebauer[1], Andreas Marek[4], Markus Rampp[4], Stefan Bauer[5], Hongxiang Li[6], Ian Baker[7], Leigh T. Stephenson[1], Baptiste Gault[1, 8,*]*

[1] Max-Planck Institut für Eisenforschung GmbH, Max-Planck-Straße 1, 40237 Düsseldorf, Germany

[2] State Key Laboratory of Powder Metallurgy, Central South University, Changsha, 410083, China

[3] Institute of Metals, College of Materials Science and Engineering, Changsha University of Science and Technology, Changsha 410114, China

[4] Max Planck Computing and Data Facility, Gießenbachstraße 2, 85748 Garching, Germany

[5] Max Planck Institute for Intelligent Systems, Max-Planck-Ring 4, 72076 Tübingen, Germany

[6] State Key Laboratory for Advanced Metals and Materials, University of Science and Technology Beijing, 100083, Beijing, China

[7] Thayer School of Engineering, Dartmouth College, Hanover, NH 03755, USA

[8] Department of Materials, Imperial College, South Kensington, London SW7 2AZ, UK

[*]Corresponding authors, yue.li@mpie.de (Y. L.); z.wang@csu.edu.cn (Z. W.); b.gault@mpie.de (B. G.)

This pdf file includes:

Supplementary Notes

Supplementary Figures

Supplementary Tables

Supplementary Notes

Supplementary Note 1. Reconstruction quality of APT data

The reconstruction reliability of APT is discussed in terms of the field evaporation simulation in APT, experimental parameters, and large-scale APT simulation.

First, we have discussed this issue at length in a previous paper [1] based on simulating the field evaporation and APT data reconstruction process, showing that the atomic neighbourhood relationships can be maintained, even in an alloy with five elements but only in the depth direction. The maximum z-direction deviation can be about 0.2 nm, but most of the atoms are still within about 0~0.1 nm. This ensures that the z-SDMs focusing on the depth-direction signal enable to represent the signature of CSRO domains.

Second, for experiments, to minimize the deviations in z, we adopted a relatively low temperature (about 50 K) and used voltage pulsing mode to ensure that the sequence of detection of the ions is controlled by the strength of the electrostatic field, which is the key to maintaining the high depth resolution [2]. The presence of readily visible poles in Fig. 1 is a good indication that this order is maintained, as it results from the shaping of the specimen from the field evaporation process itself. Moreover, the cross-species Fe-Al elemental pair was not analysed to avoid possible biases arising from differences in evaporation fields affecting the spatial resolution [3].

Finally, we synthesised large-scale APT datasets by assuming that the maximum atoms shift in the lateral and depth directions are set as 5th and 1st (0.14 nm) nearest neighbour distances, respectively. This simulates the above physical-based condition. Our ML model can distinguish B_2 domains from the BCC matrix in terms of spatial distributions (PCC>0.9), morphology, and size distributions (PCC=0.62). It is similar to DO_3 domains.

All in all, ML-APT makes optimal use of the highest-quality, near-atomic resolution of APT, combined with its high elemental analytical ability. Thereby, this enables us to precisely reveal the morphology and size distributions of multiple types of (non-)statistical CSRO, in 3D.

Supplementary Note 2. The generalizability of our ML-APT model and the diversity of simulated data

The generalizability of our model and data diversity are discussed in terms of the range of the parameters for simulated data, the visualisation of the distribution of simulated data, and the application of the recognition model of Fe-Al alloys on Fe-Ga alloys.

First, as listed in Supplementary Table 1, the main parameters for simulating specific structures under different APT conditions are varied across a wide range of parameters. For both the lateral and depth resolutions, the defined range includes the reported values that are encountered in experimental APT data. Worse resolutions, i.e., leading to a loss of the capability to image atomic planes, were not considered because generating featureless patterns would be meaningless for training the ML model. Our ML model generally classifies these noisy and featureless patterns as the BCC matrix to avoid bias from low-quality z-direction signals (see Supplementary Fig. 9). The simulated range of detection efficiency contains values for commercial APT apparatuses (0.35, 0.52, and 0.8) and includes worse conditions (e.g., 0.2). We even considered the effect of local peak shifts (-0.06~0.06nm) in the simulated SDMs database, which could reflect slight distortions arising from, e.g., the evolution of the projection parameters in the reconstruction model [4,5]. We tested the larger data size via either refining the original parameter range or extending this range slightly, but the performance of the model did not change substantially.

We further visualised the distribution of simulated data using Principal Component Analysis (PCA) and t-distributed Stochastic Neighbour Embedding (t-SNE), as shown in Supplementary Fig. 12. Different colours correspond to different structures distributed in low-dimension spaces. Both two methods suggest a good diversity of simulated data. The 1D signature of CSRO, z-SDM, is not too complex, and it is hence relatively easier to reconcile the diversity of configurations using only thousands of data points as compared to 2D images.

Finally, for the Fe-Al system with the possible occurrence of CSRO, the current model can work well for these varied compositions. This is because only the peak amplitudes in the z-SDMs will change but not the peak positions. This is also expected to work because other crystal structures are not predicted in the Fe-Al system, as per the phase diagram [6]. The same

model also was applied to the Fe-Ga system to make predictions and the results match well with previous TEM work.

Supplementary Figures and Tables

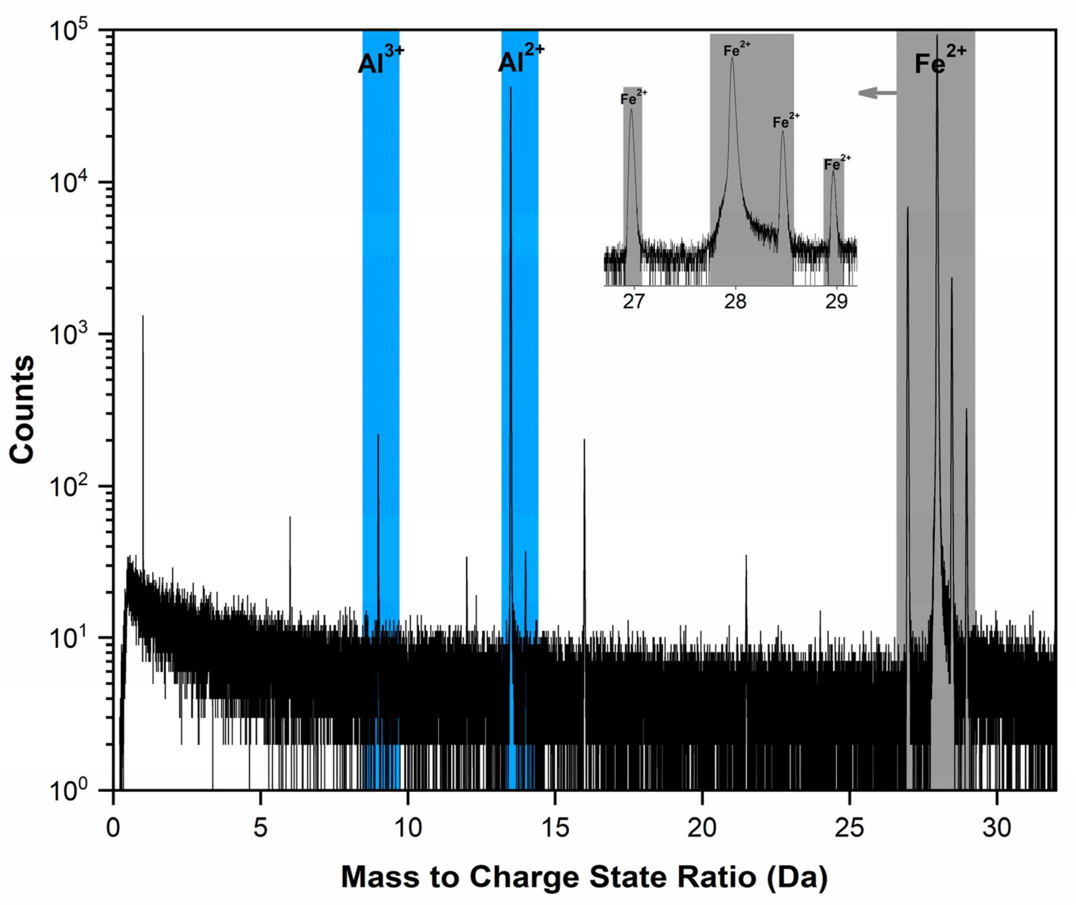

Supplementary Fig. 1 Atom probe mass spectra (voltage mode) of Fe–18Al (at.%) alloy annealing at 523 K for 14 days.

The peak at 27 Da was assigned to $^{54}Fe^{2+}$ according to the bulk composition analysis (Supplementary Table 2). No Fe^{1+} and Fe^{3+} peaks were found regardless of single or multiple hit map. There is no peak after 30 Da. AlH was approximately regarded as Al due to original Al adsorbed by H.

a **Details of the used 1D convolutional neural network**

Layer type	Specifications
1D CNN layer	Kernel size: 10; 64 filters; stride: 1; padding: Same; Relu
Dropout layer	Rate: 0.5
1D Max pooling layer	Pool size: 2; stride: 2; Valid
Fully connected layer	Size: 256
Output layer	Size: 3

b

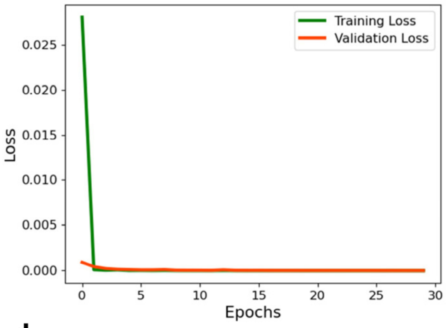

c **d**

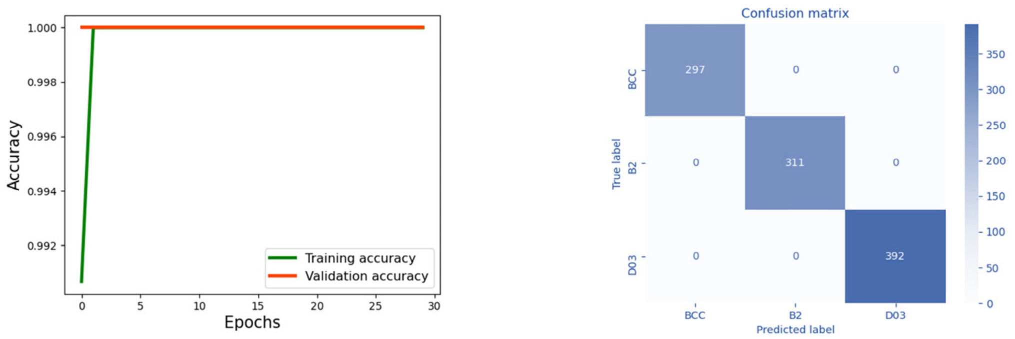

Supplementary Fig. 2 Details of the optimized 1D CNN structure and the training, validation, and test I results.

a The 1D CNN configuration. **b, c** The evolutions of the training and validation losses and accuracies. **d** The confusion matrix of the test results using the 10% simulated data. Note that the random forest algorithm exhibited almost the same training, validation, and test I performance (not shown here).

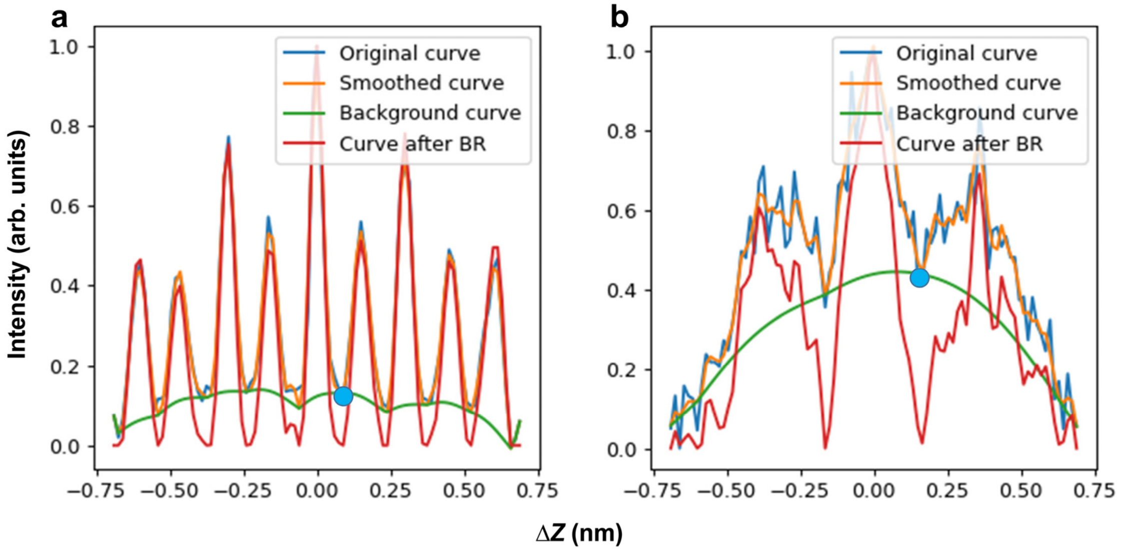

Supplementary Fig. 3 Typical experimental z-SDMs before and after data pre-processing. **a**, **b**, Curves with BCC matrix and CSRO signals, respectively. The original curve was initially smoothed using a Savitzky–Golay filter, and then its background was subtracted (Methods). BR refers to removing background. The blue circles highlight the sites of the first local minimum after x-axis is above 0, which was taken as a criterion to stop the iterative background estimation.

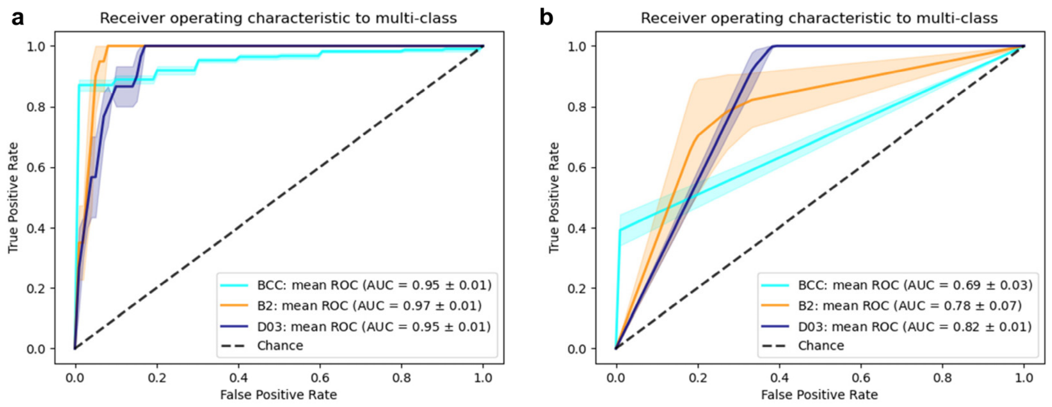

c

		Predicted condition			
	Total	D03<br>32	Non-D03<br>116		
Actual condition	D03<br>6	6	0	TPR=1	FNR=0
	Non-D03<br>142	26	116	FPR=0.18	TNR=0.82

d

		Predicted condition			
	Total	B2<br>23	Non-B2<br>125		
Actual condition	B2<br>4	4	0	TPR=1	FNR=0
	Non-B2<br>144	19	125	FPR=0.13	TNR=0.87

Supplementary Fig. 4 ROC analysis of the 1D CNN and random forest algorithms obtained using 148 experimental data.

a, b ROC curves of the 1D CNN and random forest with uncertainties represented by the standard deviations from the predictions obtained by the five models, respectively, corresponding to three kinds of structures. The relevant AUC values and standard deviations are given. **c, d** Relevant confusion matrix of $D0_3$ and B_2 using the CNN, respectively. TPR, TNR, FNR and FPR represent true positive rate, true negative rate, false negative rate, and false positive rate. The nature of the sparse distribution of CSRO in BCC leads to the imbalance of labels. This pushed us to choose the ROC and AUC, which are insensitive to the distribution of data [7].

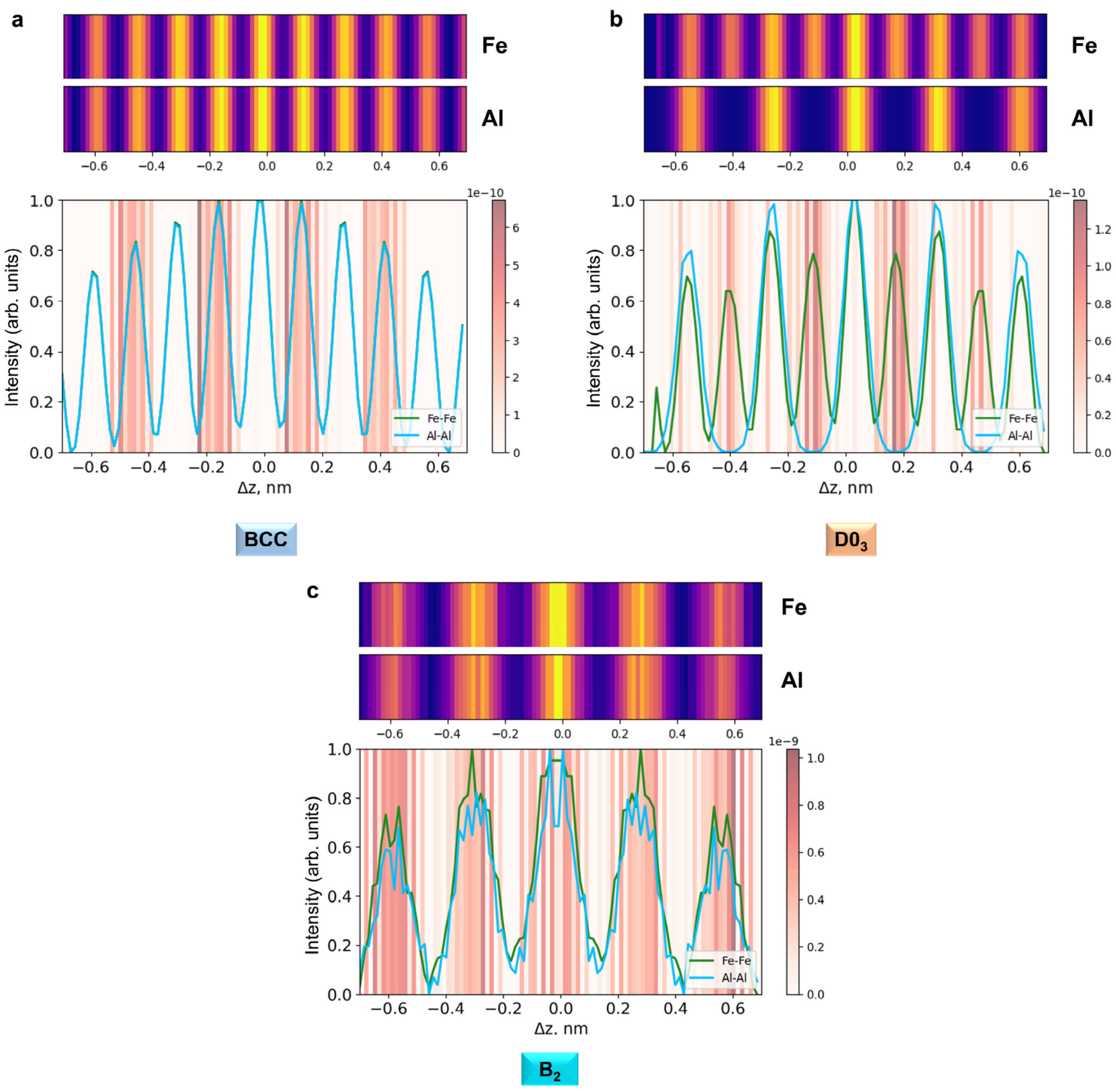

Supplementary Fig. 5 Visualization of the obtained CNN model on three classes of z-SDMs via gradient-weighted class activation mapping.

a BCC. **b** D0$_3$. **c** B$_2$. The heatmap of z-SDMs of Fe-Fe and Al-Al pairs is given using a plasma colour map. The heatmap corresponding to gradient-weighted class activation mapping is plotted using a red colour map. The higher level of red colour indicates more attention. For the two formers, the model is looking at whether there are peaks at the zones close to the ΔZ with ±0.144 and ±0.432 nm. For the B$_2$, the model focuses on the zones close to the ΔZ with 0, ±0.288 or ±0.576 nm.

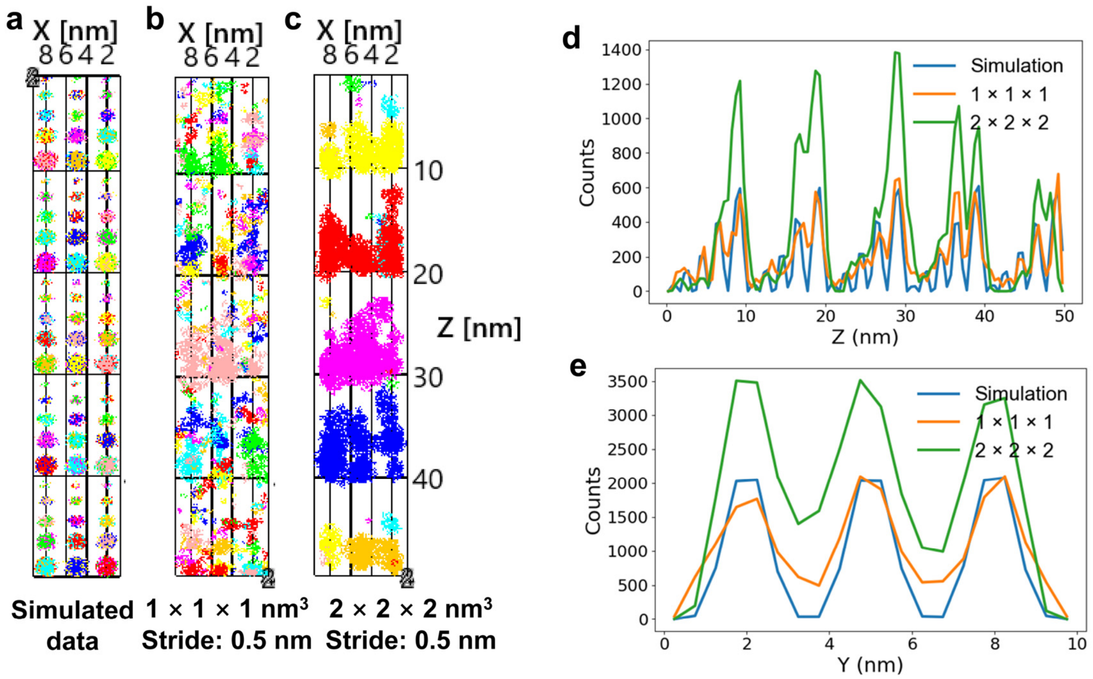

f

Data in d	1x1x1 vs simulation	2x2x2 vs simulation
PCC	0.85	0.69

Data in e	1x1x1 vs simulation	2x2x2 vs simulation
PCC	0.96	0.92

Supplementary Fig. 6 Test of the obtained ML-APT recognition model in large-scale Fe-Al APT simulation with $D0_3$-CSRO domains.

a Simulated $D0_3$-CSRO APT data. **b, c** Recognized CSRO domains via the proposed recognition model using 1 × 1 × 1 and 2 × 2 × 2 nm^3 scanning cubes, respectively. **d, e** Distributions of counts of atoms in **a-c** along Z and Y directions, respectively. **f** The Pearson's correlation coefficients (PCC) in **d, e** are listed.

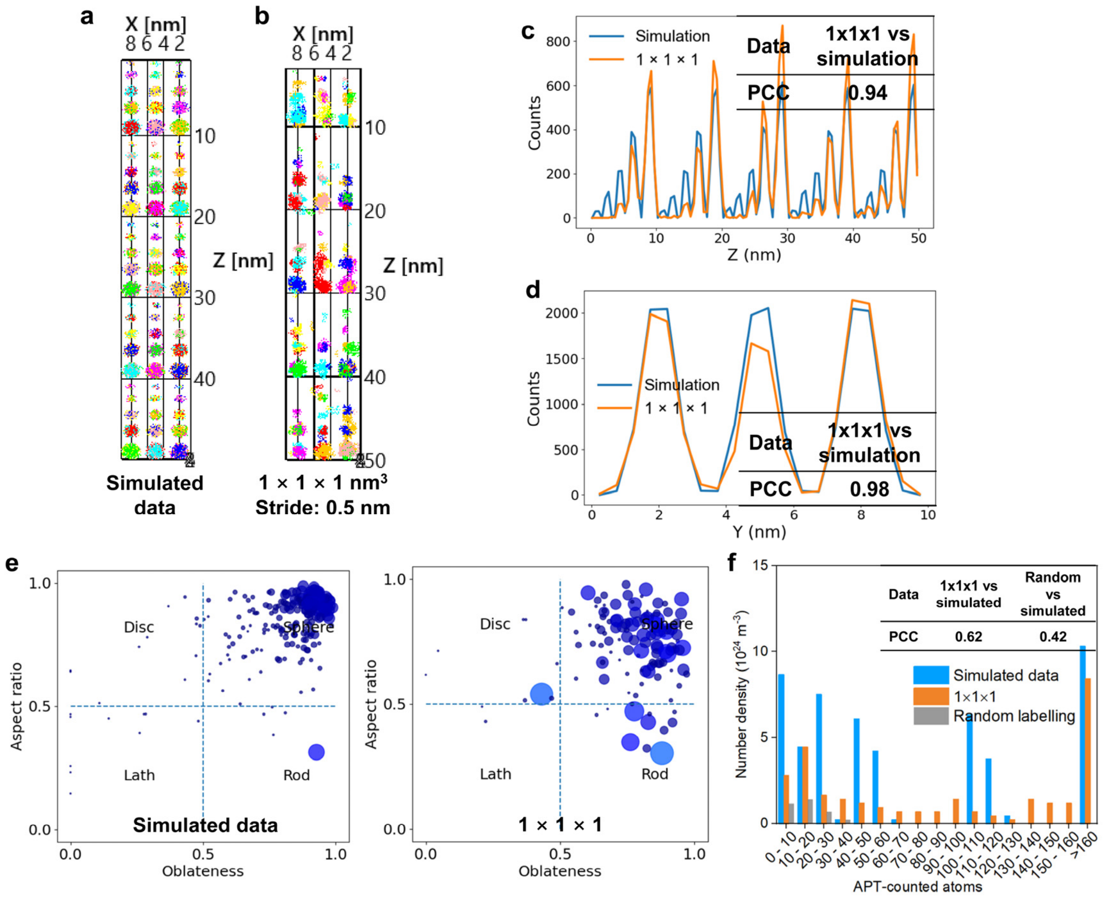

Supplementary Fig. 7 Test of the obtained ML-APT recognition model in large-scale Fe-Al APT simulation with B_2-CSRO domains.

a Simulated B_2-CSRO APT data. **b** Recognized CSRO domains via the proposed recognition model using 1 × 1 × 1 nm^3 scanning cubes. **c, d** Distributions of counts of atoms in **a** and **b** along Z and Y directions, respectively. The PCC values are listed in the inserted tables. **e** Morphology maps of the simulated CSRO domains and detected ones via the proposed model using 1 × 1 × 1 nm^3 scanning cubes. The size and colour of one circle denote the number of atoms within one domain. **f** Number densities versus CSRO size range corresponding to simulated and recognized CSRO domains. The result from the chemically-randomized dataset (Methods) is compared, and the PCC values are listed in the inserted table.

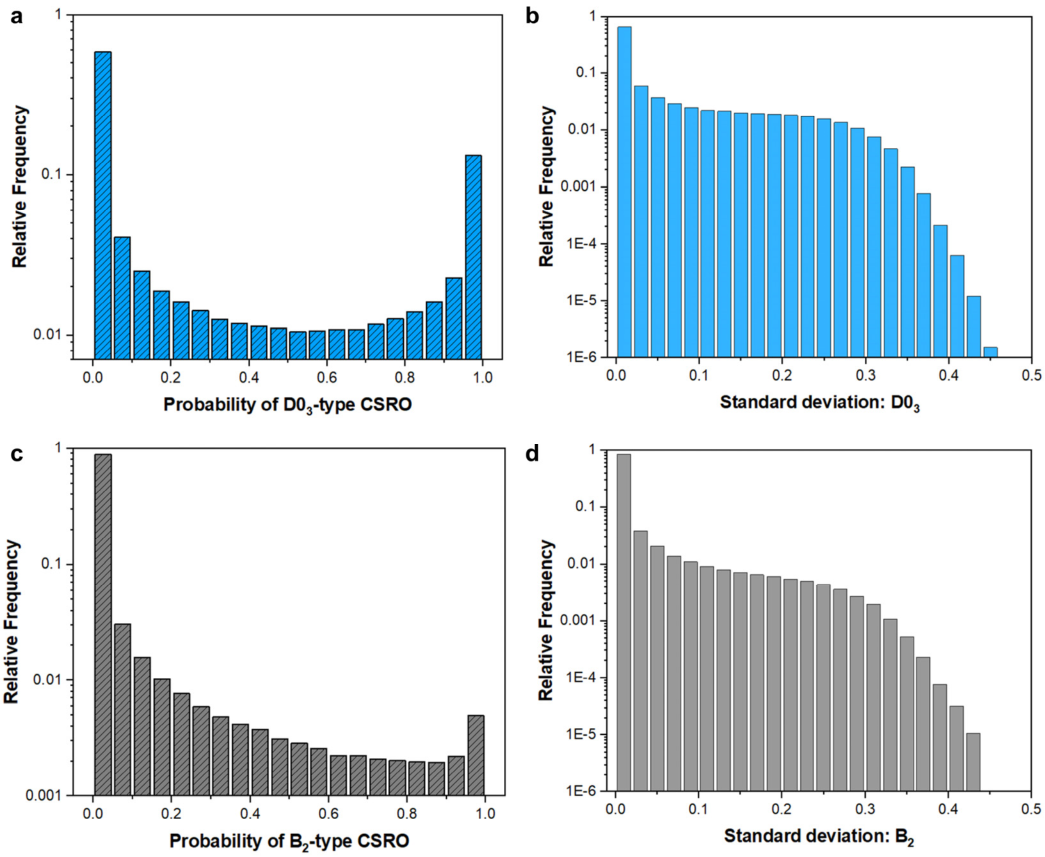

Supplementary Fig. 8 Frequency distributions of the predicted two types of CSRO structures probabilities and uncertainties of 653944 1-nm^3 voxels generated from APT data shown in Fig. 1a.

a, c Distributions of the predicted $D0_3$-CSRO and B_2-CSRO structures probabilities. 1 represents the highest $D0_3$-CSRO and B_2-CSRO structures probabilities. **b, d** Distributions of the uncertainties (standard deviations) obtained from five-fold cross validation of the $D0_3$-CSRO and B_2-CSRO structures.

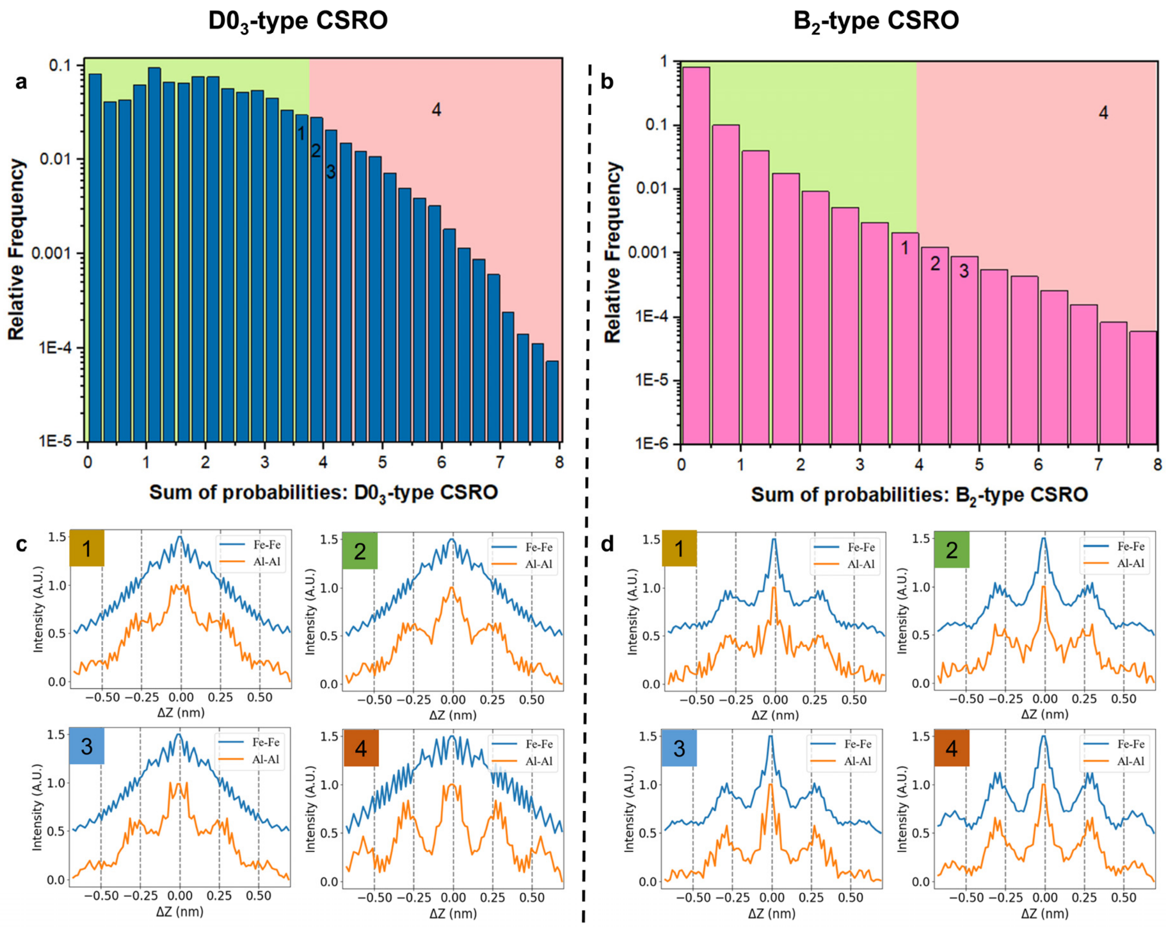

Supplementary Fig. 9 Predictions of two kinds of CSRO structures in Fe-18Al data.

a, b Frequency distributions of two kinds of CSRO structure probabilities of the 0.5-nm^3 voxels ($P_{0.5}$) obtained from Fig. 1**a**. The line divided by different colours indicates the determined threshold for classification. **c, d** The z-SDMs generated by the data corresponding to zone 1, 2, 3, and 4 in **a** and **b** (before data pre-processing).

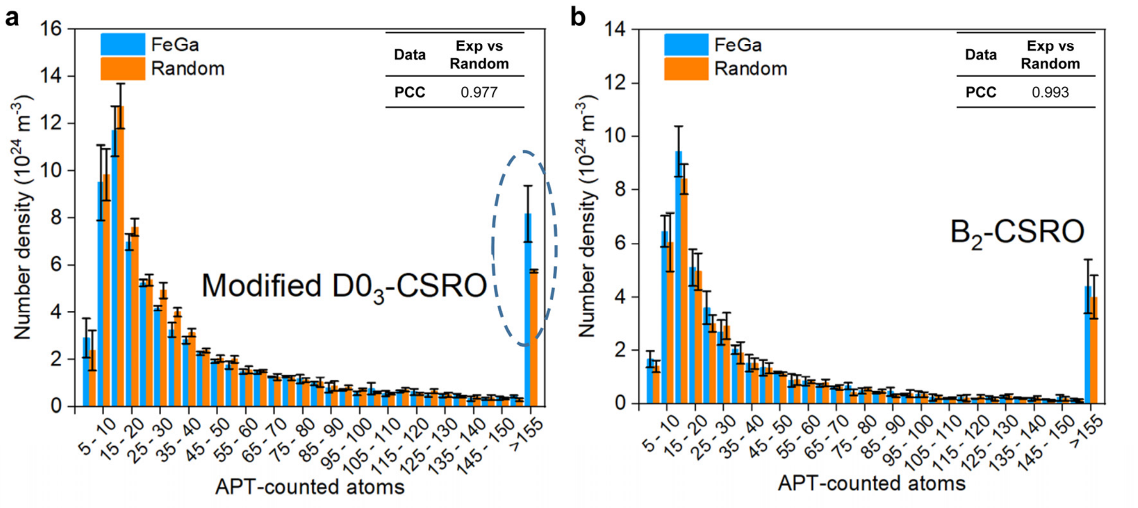

Supplementary Fig. 10 Quantitative CSRO distributions in a Fe-19 at.% Ga alloy.

a, **b** Number density distributions of different sizes of modified $D0_3$- and B_2-CSRO, respectively. The result from the chemically-randomized dataset is compared with the PCC values. All error bars (standard deviations) were obtained by three measurements.

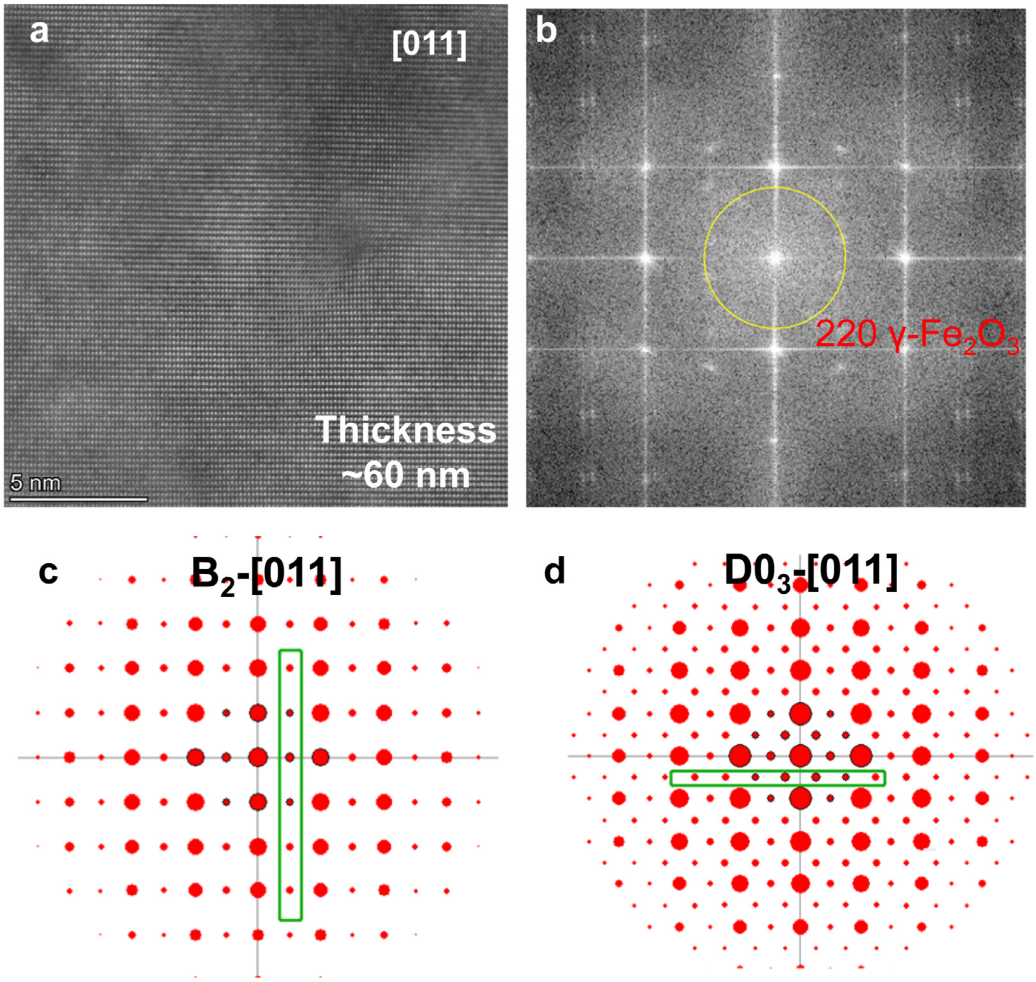

Supplementary Fig. 11 Characterization of CSRO based on STEM analysis in Fe-18Al alloys annealing at 523 K for 14 days from the [011] zone axis.

a High-angle annular dark field scanning transmission electron microscopy images from the [011]. **b** Corresponding fast Fourier transform pattern. **c, d** Simulated electron diffraction pattern of B_2 and $D0_3$ from the [011], respectively. The diffraction patterns except BCC-Fe do not match well with $D0_3$ and B_2 and the sharp points indicate that an ordered structure has been formed with a possible phase index of γ-Fe_2O_3. The oxide films inevitably exist on TEM specimens which can contribute diffractions [8], while this negative effect can be easily avoided when analyzing APT data.

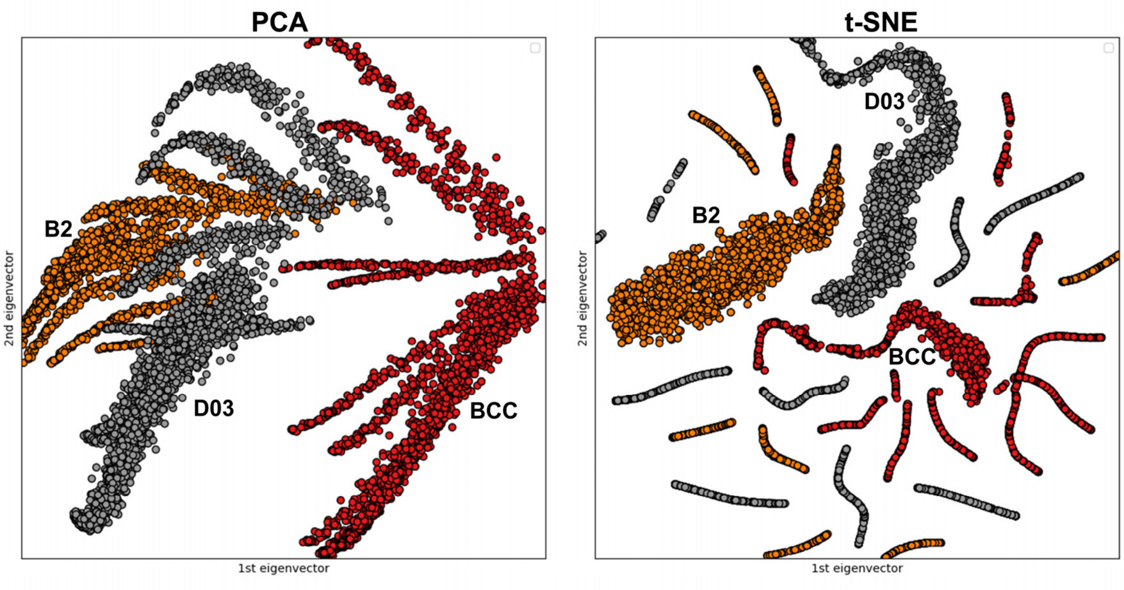

Supplementary Fig. 12 PCA and t-SNE analysis of the simulated data.

The red, grey, and orange colours represent BCC, $D0_3$, and B_2, respectively.

Supplementary Table 1 Parameters for building the crystal structure library and generating the corresponding z-SDMs bank.

Note that "σ" represented the standard deviation of the Gaussian function.

Category	Number of z-SDMs	$\boldsymbol{\sigma_x = \sigma_y}$, nm	$\boldsymbol{\sigma_z}$, nm	Detect efficiency	Peak shift, nm
BCC	3000	0.2~0.8	0.02~0.05	0.2~0.7	-0.06~0.06
D03	4000	0.2~0.8	0.01~0.05	0.2~0.7	-0.06~0.06
B_2	3000	0.2~0.8	0.03~0.06	0.2~0.7	-0.06~0.06

Supplementary Table 2 Bulk composition of APT data of Fe-18Al (at.%) alloy corresponding to the mass spectra in Supplementary Fig. 1.

The composition related to further analysis refers to the assignment of the peak at 27 Da to Al^{+}.

Element	Peak decomposed, at.%	Composition related to further analysis, at.%
Fe	80.619	77.414
Al	17.116	22.327
C	0.030	0.029
O	0.103	0.102
H	0.132	0.129

Supplementary References

1 Gault, B. *et al.* Reflections on the spatial performance of atom probe tomography in the analysis of atomic neighbourhoods. *Microsc. Microanal.* 28, 1116-1126 (2022).

2 Jenkins, B. M. *et al.* Reflections on the Analysis of Interfaces and Grain Boundaries by Atom Probe Tomography. *Microsc. Microanal.* 26, 247-257 (2020).

3 Vurpillot, F., Bostel, A., Cadel, E. & Blavette, D. The spatial resolution of 3D atom probe in the investigation of single-phase materials. *Ultramicroscopy* 84, 213-224 (2000).

4 Loi, S. T., Gault, B., Ringer, S. P., Larson, D. J. & Geiser, B. P. Electrostatic simulations of a local electrode atom probe: The dependence of tomographic reconstruction parameters on specimen and microscope geometry. *Ultramicroscopy* 132, 107-113 (2013).

5 Gault, B. *et al.* Dynamic reconstruction for atom probe tomography. *Ultramicroscopy* 111, 1619-1624 (2011).

6 Palm, M., Stein, F. & Dehm, G. Iron Aluminides. *Annual Review of Materials Research* 49, 297-326 (2019).

7 Fawcett, T. An introduction to ROC analysis. *Pattern Recognition Letters* 27, 861-874 (2006).

8 Sasidhar, K. N. *et al.* Understanding the protective ability of the native oxide on an Fe-13 at% Cr alloy at the atomic scale: A combined atom probe and electron microscopy study. *Corros. Sci.* 211, 110848 (2023).
